# Therapeutic Efficacy of Hyperbaric Oxygen in Central Retinal Artery Occlusion: A Systematic Review and Meta-Analysis

**DOI:** 10.3390/jcm15093530

**Published:** 2026-05-05

**Authors:** Hani Basher ALBalawi, Moustafa S. Magliyah, Naif M. Alali, Mohammed M. Alshehri, Abdullah Alhewiti, Faisal Almarek, Ibrahim Shajry, Mohammad A. Hazzazi, Yousef A. Alotaibi

**Affiliations:** 1 Division of Ophthalmology, Department of Surgery, Faculty of Medicine, University of Tabuk, Tabuk 47512, Saudi Arabia; nmalali@ut.edu.sa; 2 Vitreoretinal Division, King Khaled Eye Specialist Hospital, Riyadh 11462, Saudi Arabia; mussam8@yahoo.com; 3 Diving and Hyperbaric Medicine Department, NEOM Hospital, Sharma 49626, Saudi Arabia; dr.help20@gmail.com; 4 Department of Family and Community Medicine, Faculty of Medicine, University of Tabuk, Tabuk 47512, Saudi Arabia; aalhewiti@ut.edu.sa; 5 Department of Ophthalmology, Faculty of Medicine, Imam Mohammad ibn Saud Islamic University, Riyadh 13317, Saudi Arabia; falmarek@gmail.com; 6 Ophthalmology Department, King Khaled University Medical City, Abha 62529, Saudi Arabia; ibrahimshajry@gmail.com; 7 Department of Surgery, Division of Ophthalmology, National Guard Hospital, Riyadh 14611, Saudi Arabia; dr.alhazzazi@gmail.com; 8 College of Medicine, King Saud Bin Abdulaziz University for Health Sciences, Riyadh 14611, Saudi Arabia; 9 Department of Ophthalmology, College of Medicine, King Khalid University, Abha 61413, Saudi Arabia; yalotaibi@kku.edu.sa

**Keywords:** central retinal artery occlusion, hyperbaric oxygen therapy, visual recovery, systematic review, meta-analysis, adverse events

## Abstract

**Background/Objectives:** Central retinal artery occlusion (CRAO) is a vision-threatening condition with limited evidence-based treatment options. Hyperbaric oxygen therapy (HBOT) has emerged as a potential intervention, but its efficacy remains debated. This systematic review and meta-analysis evaluated the therapeutic efficacy and safety of HBOT in CRAO. **Methods:** Relevant studies were identified across seven databases using optimized Boolean and MeSH-based strategies. Eligible studies evaluated HBOT in CRAO and reported visual or safety outcomes. Extracted data included demographics, intervention details, treatment timing, visual acuity outcomes, and adverse events. Risk of bias was assessed using ROBINS-I. Visual acuity outcomes were standardized to logMAR whenever directly reported or convertible, and subgroup analyses were stratified by HBOT initiation time (<12 h vs. >12 h), study type, and baseline visual severity when reported. A random-effects model was used, and pooled estimates were expressed as odds ratios (ORs) with 95% confidence intervals (CIs). **Results:** Twelve studies were included. The pooled efficacy analysis favored HBOT (OR = 0.47; 95% CI: 0.26–0.87; *p* = 0.02), although heterogeneity was substantial (Tau^2^ = 0.64; I^2^ = 78%). Stratified synthesis showed that studies in which HBOT was initiated within 12 h consistently reported greater visual improvement, whereas delayed or variably timed treatment showed attenuated and inconsistent benefits. After outcome harmonization, studies reporting logMAR-compatible data generally demonstrated clinically relevant visual improvement, while adverse-event rates did not differ significantly between HBOT and non-HBOT groups (OR = 0.70; 95% CI: 0.43–1.16; *p* = 0.17; I^2^ = 0%). **Conclusions:** HBOT appears most beneficial when initiated early, particularly within the first 12 h. However, heterogeneity in treatment timing, study design, and baseline severity reporting limits the certainty of these results and supports the need for standardized outcome reporting and protocol-driven prospective studies.

## 1. Introduction

Central retinal artery occlusion (CRAO) is a serious ocular emergency, resulting in sudden severe loss of vision and often total irreversible blindness [1]. It occurs due to a sudden blockage of the central retinal artery, which is the principal artery supplying oxygen-rich blood to the retina. The incidence of CRAO is relatively low, ranging from 1 to 10 per 100,000 individuals annually, but because its onset is so rapid and the retina has such a high metabolic demand, the effect can be dramatic [2]. This emphasizes the need for effective therapeutic strategies for this condition, as retinal neurons may suffer irreversible damage within minutes to hours unless adequate treatment is received in time [3].

The pathology of CRAO is closely related to systemic conditions, including atherosclerosis, hypertension, diabetes mellitus, and heart disease, leading either to embolism or thrombosis related to central retinal artery occlusion [4]. As it is related to cardiovascular risk factors, the pathophysiology of CRAO overlaps with cerebrovascular accidents, demanding a multidisciplinary approach to managing affected patients. Unlike ischemic strokes, no standardized treatment protocol has been established for CRAO; therefore, the clinical prognosis remains indeterminate [5,6,7].

Some of the conventional remedies for CRAO are ocular massage, anterior chamber paracentesis, systemic and topical intraocular pressure-reducing agents, and intravenous thrombolysis [8]. However, evidence supporting these treatments is often tenuous because CRAO is an infrequent phenomenon, and conducting large-scale clinical studies of such a rare disorder is challenging. Furthermore, the very short therapeutic window of time available, generally less than 24 h after onset of symptoms, does not allow for timely initiation of any of these treatments in routine clinical practice [9]. The search for effective, time-sensitive interventions capable of ameliorating ischemic damage in CRAO has, consequently, driven an increasing interest in hyperbaric oxygen therapy [10].

HBOT is the administration of 100% oxygen at pressures greater than atmospheric pressure, which enhances the delivery of oxygen to hypoxic tissues via plasma [9]. This effect should, therefore, be applied to the treatment of CRAO, since oxygen partial pressure inside the retinal artery and the surrounding tissue can increase and thus prevent ischemic damage. The primary rationale for the use of HBOT in CRAO is theoretical; it is based on improved oxygenation of the retina, resulting in increased perfusion and an increased window before spontaneous reperfusion [11]. Apart from the advantages listed above, HBOT has been used successfully in other ischemic conditions such as decompression sickness and ischemic wounds, forming the basis for its consideration as a therapeutic modality in CRAO [12].

Despite all these theoretical advantages, the clinical benefit of HBOT in CRAO remains controversial. Previous studies, conducted mainly in the form of case reports and small case series, suggest an apparent benefit of HBOT in the recovery of vision if applied early [10,11,12]. However, these studies have significant variability in patient selection, treatment protocols, and outcome measures, resulting in conflicting conclusions. In addition, the rarity of CRAO has significantly limited the opportunity to conduct large-scale randomized controlled clinical trials. Instead, observational studies have had to rely on evidence synthesis to provide information.

While the evidence on HBOT for CRAO is potentially viable for real-world treatment, the practical applicability and generalization of these findings remain unclear. Hence, there is a need for a systematic and comprehensive review of the available evidence to aid better clinical decision-making. Therefore, the central aim of our systematic review and meta-analysis is to critically appraise and synthesize the existing evidence on the efficacy and safety of HBOT as an adjunct treatment in the management of CRAO.

## 2. Materials and Methods

### 2.1. Research Question and Focus

The primary research question was, what are the efficacy and safety of HBOT as a treatment modality for CRAO, in terms of improving visual acuity, enhancing reperfusion rates, and minimizing adverse events compared to standard treatments or no treatment?

### 2.2. Eligibility Criteria

The inclusion criteria consisted of human studies that included subjects suffering from CRAO who received hyperbaric oxygen therapy as part of their treatment procedure without any regard to their age, gender or geographical location. Studies that compared HBOT to no treatment, standard treatment, or another treatment modality for CRAO were included as long as they provided information on at least one of the outcome measures—such as changes in visual acuity, reperfusion effects, or adverse events. Cohort studies, case–control studies, and case series comprising at least three patients were also included. Studies not using HBOT, lacking information about the intervention used, failing to provide useful outcomes data, single cases, reviews, editorials, conference abstracts, thesis papers, seminars, animal studies and in vitro studies were excluded. The search was limited to full-text English-language articles available up until October 2024. RCTs were not considered since no studies fitting the inclusion criteria were found among those employing this design. The main reasons for such a finding could be associated with the rare occurrence of CRAO, narrow time window for effective HBOT, and difficulties with randomization given the scope of this urgent procedure.

### 2.3. PECOS Protocol and PRISMA Compliance (See Supplementary Materials)

For this systematic review, the PECOS protocol was used to design and create a well-defined and structured review. The population (P) studied consisted of all patients diagnosed with CRAO, irrespective of their age, sex, or location. Treatment exposure (E) was defined as receiving HBOT. The treatment protocols and duration included all of these variations. The comparator (C) was nothing, ocular massage, anterior chamber paracentesis, intravenous thrombolysis, or any other form of therapeutic intervention given for CRAO. The results (O) measured included improvement in visual acuity, rate of reperfusion, and any adverse events reported post treatment. The study design (S) included cohort studies, case–control studies, and case series with a minimum of three participants. This protocol adhered to PRISMA [13] in terms of ensuring transparency and comprehensiveness in the selection process, screening, and reporting procedure, and the protocol was registered before the commencement of the review in the PROSPERO database (CRD42024625513 https://www.crd.york.ac.uk/PROSPERO/view/CRD42024625513 (accessed on 1 October 2024). More information can be found in the Supplementary Materials.

### 2.4. Search Strategy

According to the search strategy developed, this study identified relevant studies sourced from across the seven major databases concerned with the evaluation of HBOT for CRAO. These included PubMed, Embase, Web of Science, Cochrane Library, Scopus, CINAHL, and ClinicalTrials.gov. The Boolean operators were employed in conjunction with MeSH to ensure all relevant studies could be efficiently retrieved. The search terms comprised the various terms “Central Retinal Artery Occlusion,” “CRAO,” “Hyperbaric Oxygen Therapy,” and “Visual Recovery.” Appropriate Boolean operators, including “AND” and “OR,” were used to integrate relevant synonyms and related terms. The search strategy was tailored to each database’s indexing system so that studies could be retrieved optimally while considering different study designs and publication types. Duplicates were removed, and the results were filtered based on the inclusion and exclusion criteria.

### 2.5. Data Extraction Protocol

Following a systematic protocol, data extraction was conducted uniformly using a predefined data extraction form. The form included information such as study characteristics, including the author, year of publication, country, and study type; patient demographics, including age and gender distribution; intervention features, including HBOT pressure, session duration, and number of sessions; comparator details; and outcomes, including changes in visual acuity, time to reperfusion, and adverse events. Additional extraction fields included sample size, follow-up period, baseline visual acuity severity, original visual acuity scale, and statistical methods. Visual acuity data were preferentially recorded as logMAR whenever directly available, while decimal and Snellen-based measures were retained for later harmonization when sufficient numeric information was provided. The process of extraction was carried out in duplicate by two independent reviewers to minimize error, and any discrepancy was resolved through consultation with a third reviewer. This approach supported qualitative synthesis, timing-based stratification (<12 h vs. >12 h), and subgroup analyses by study type and baseline severity.

### 2.6. Bias Assessment Protocol

The non-randomized studies included in this study were evaluated for their methodological quality using ROBINS-I [14]. The tool was applied in seven predefined domains. Domain D1 (“Bias due to confounding”) was used to test for confounders, i.e., whether baseline variables, such as age, symptom duration, initial visual acuity levels, disease severity, presence of vascular risk factors, or other concomitant treatments might lead to biased results regarding the effects of HBOT. Domain D2 (“Bias in selection of participants into the study”) tested the risk of selecting participants in ways that led to the systematic introduction of differences between groups, especially those related to prognosis, therapy timing, or clinical indications. Domain D3 (“Bias in classification of interventions”) tested the clarity of HBOT exposure and comparison therapies, with special emphasis on defining and classifying the treatments properly (identification of treatment, types of protocols, treatment initiation times). Domain D4 (“Bias due to deviations from intended interventions”) checked the accuracy of following the protocol throughout the study—any co-intervention, deviations from the intended procedure, and management issues were considered. Domain D5 (“Bias due to missing data”) considered the completeness of outcome data reporting. The following aspects were of particular interest: any losses to follow-up; visual acuity assessment failures; and missing adverse-event reports. Domain D6 (“Bias in measurement of outcomes”) considered reporting of outcome measures, with the focus on objective measurement of visual acuity changes, reperfusion-related outcomes, and adverse events. Domain D7 (“Bias in selection of the reported result”) concerned the problem of selecting only statistically significant results from analyses and time points. Each of the domains was evaluated individually, and overall ROBINS-I judgments were provided for each study.

### 2.7. Meta-Analysis Protocol

The meta-analysis was performed using RevMan 5 version 5.4.1, thereby combining the treatment effects of HBOT among patients with CRAO. Forest plots illustrating odds ratios (ORs) for dichotomous outcomes such as unfavorable visual outcomes and adverse events were generated. For continuous visual outcomes, all compatible measures were interpreted on a logMAR scale to reduce metric inconsistency across studies. A random-effects model was adopted because heterogeneity was expected across patient populations, HBOT protocols, comparator interventions, and treatment timing. Prespecified subgroup analyses were performed according to HBOT initiation time (<12 h vs. >12 h), study type (prospective vs. retrospective/case series), and baseline visual severity when adequately reported. Studies with mixed timing windows or non-convertible visual metrics were synthesized narratively within these strata. Ninety-five percent confidence intervals (CI) were calculated, and the I^2^ statistic was used to investigate heterogeneity among the included studies.

## 3. Results

**Figure 1:**
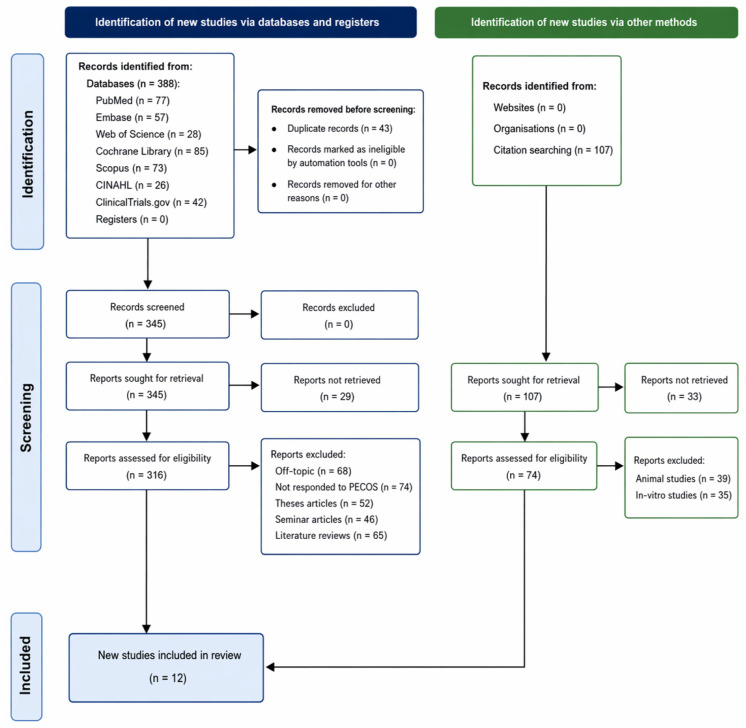
Representation of the study selection process for this review.

Initially, 388 records were retrieved from the databases PubMed, 77; Embase, 57; Web of Science, 28; Cochrane Library, 85; Scopus, 73; CINAHL, 26; and ClinicalTrials.gov, 42 (Figure 1). After excluding the duplicate 43 records, a further 345 records were screened. The reports of 29 of these studies could not be retrieved. The remaining 316 records were assessed for eligibility, with 205 excluded for reasons such as being off-topic (68), not adhering to PECOS criteria (74) and being thesis articles (52), seminar articles (46), and literature reviews (65). An additional 74 records identified through citation searching were also assessed, excluding 39 animal studies and 35 in vitro studies. Finally, 12 studies [15,16,17,18,19,20,21,22,23,24,25,26] were included in the review.

### 3.1. Study Design and Technical Characteristics

As elucidated through Table 1 and Table 2, the included studies represented retrospective analyses, case series, and prospective investigations, with sample sizes ranging from 13 (Lopes et al. [19]) to 144 (Rozenberg et al. [23]). Participant age was generally in the late sixth to seventh decade, although somewhat younger cohorts were reported in a few studies such as Yip et al. [26]. Male-to-female ratios varied substantially, and the interval from symptom onset to HBOT ranged from very early initiation (<8 h) to broad or delayed windows extending beyond 24 h. For the present synthesis, these studies were additionally examined according to initiation-time strata, original visual acuity metric, and availability of baseline severity data in order to support more structured subgroup interpretation.

### 3.2. Standardized Visual Acuity Outcomes

Visual acuity was originally reported using heterogeneous metrics, including decimal visual acuity, Snellen-line change, best-corrected visual acuity categories, and logMAR. For cross-study comparison, logMAR was used as the preferred interpretive scale whenever directly reported or reasonably derivable. Under this standardized framework, studies such as Chiabo et al. [17], Maldonado et al. [20], Rozenberg et al. [23], Williamson et al. [25], and Yip et al. [26] showed improvements ranging from approximately −0.20 to −0.74 logMAR or equivalent directional gains. Beiran et al. [16] and Menzel-Severing et al. [21] also favored HBOT, although their outcomes were reported in decimal acuity and Snellen-line change, respectively, which constrained direct quantitative pooling.

By contrast, Kalaw et al. [18] and Rosignoli et al. [22] reported non-significant changes despite use of BCVA/logMAR-oriented endpoints, indicating that outcome harmonization alone did not account for between-study variability. Rather, standardized interpretation suggested that timing of HBOT initiation, baseline visual severity, and study design were more plausible sources of heterogeneity than the visual acuity scale itself. Accordingly, logMAR standardization improved interpretability of effect direction and magnitude, but it did not eliminate the substantial clinical and methodological heterogeneity across the evidence base.

### 3.3. Timing Stratification and Protocols

Treatment initiation emerged as one of the strongest effect modifiers. When studies were stratified by timing, those initiating HBOT within 12 h of symptom onset generally demonstrated more consistent visual recovery, whereas studies with treatment windows extending beyond 12 h or reported as variable/broad showed mixed results. Beiran et al. [16] and Menzel-Severing et al. [21], both representing very early intervention, reported a clear benefit, while Rosignoli et al. [22] (median 18.27 h) and studies with delayed or ill-defined initiation patterns showed minimal or non-significant gains. Protocol heterogeneity persisted across strata, with most studies using 2.4–2.8 ATA for 60–90 min; however, the direction of the findings suggested that timing exerted a stronger influence on outcome than modest variation in chamber pressure or session length. Differences in session frequency, such as the twice-daily regimen used by Chiabo et al. [17], may have contributed additional variability.

### 3.4. Safety and Adverse Events

Rare adverse events mostly were mild in nature. Barotrauma of a minor type was observed in Chiabo et al. [17]. The only adverse effects mentioned by Rosignoli et al. [22] are rare and related to seizures and barotrauma. According to Williamson et al. [25], some hemotympanum and anxiety appeared, but the adverse events were not significant enough to stop treatment. In general, most of the articles stated that no significant adverse effects were seen; for instance, Beiran et al. [16] and Lopes et al. [19].

### 3.5. Statistical and Methodological Evaluations

Different statistical techniques were used across the included studies, ranging from chi-square and *t*-tests to logistic regression and mixed-effects models. Menzel-Severing et al. [21] and Maldonado et al. [20] applied regression models to control for confounders, which strengthened the reliability. However, heterogeneity in study designs and sample sizes limited the possibility of direct comparisons and underlined the need for standardized methodologies.

### 3.6. HBOT Efficacy and Adverse Events Observed

**Figure 2:**
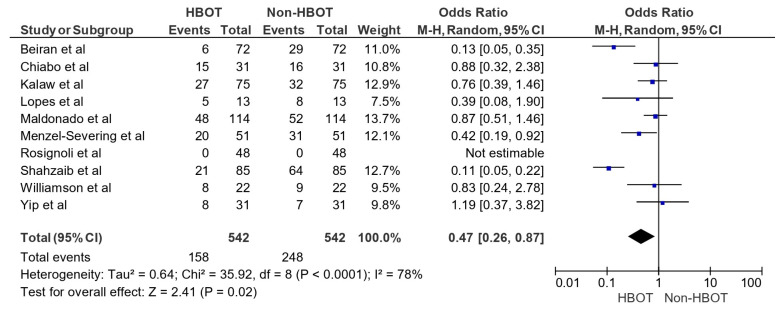
Efficacy of HBOT in management of CRAO [16,17,18,19,20,21,22,24,25,26].

In Figure 2, the pooled OR for the efficacy of HBOT was 0.47 (95% CI: 0.26–0.87; *p* = 0.02), indicating a statistically significant reduction in unfavorable outcomes in the HBOT group compared to the non-HBOT group and demonstrating the efficacy of HBOT in the management of CRAO. Individual study estimates varied, with Beiran et al. [16] reporting an OR of 0.13 (95% CI: 0.05–0.35) and Shahzaib et al. [24] showing the lowest OR of 0.11 (95% CI: 0.05–0.22), while Yip et al. [26] reported an OR of 1.19 (95% CI: 0.37–3.82), suggesting no clear benefit in that study. Significant heterogeneity was observed (Tau^2^ = 0.64; Chi^2^ = 35.92, df = 8, *p* < 0.0001; I^2^ = 78%), highlighting variability among the studies.

**Figure 3:**
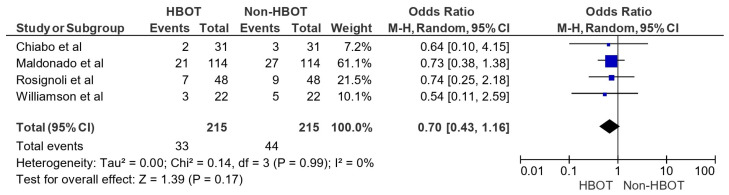
Adverse events recorded (HBOT vs. non-HBOT) [17,20,22,25].

In Figure 3, the pooled OR for adverse events in the HBOT group versus the non-HBOT group was 0.70 (95% CI: 0.43–1.16; *p* = 0.17), indicating no statistically significant difference in adverse event incidence. Individual studies, such as Maldonado et al. [20] and Rosignoli et al. [22], showed ORs of 0.73 (95% CI: 0.38–1.38) and 0.74 (95% CI: 0.25–2.18), respectively, supporting this finding. Heterogeneity was low (Tau^2^ = 0.00; Chi^2^ = 0.14, df = 3, *p* = 0.99; I^2^ = 0%), suggesting consistency across studies.

### 3.7. Bias Levels Observed

**Figure 4:**
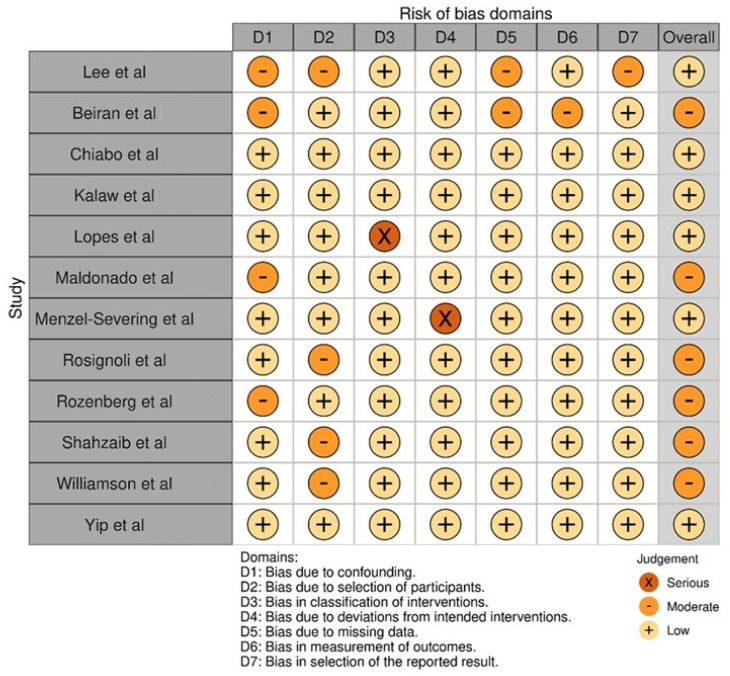
Bias assessment across the studies included in the review [15,16,17,18,19,20,21,22,23,24,25,26].

The bias assessment of the included studies, performed with the ROBINS-I tool (Figure 4), showed heterogeneity in terms of risk levels across the domains. Most of the included studies had low risk in more than one domain, signifying methodological strength. Beiran et al. [16], Chiabo et al. [17], and Shahzaib et al. [24] had low risk in all domains, which indicated good internal validity. Kalaw et al. [18] and Lopes et al. [19] had moderate risk in some domains, like D3 (classification of interventions) and D6 (measurement of outcomes), due to the constraints in the study design. The moderate risk in D2 (confounding) and D6 was reported by Maldonado et al. [20] and by Rosignoli et al. [22]. There were insufficient adjustments made regarding confounding variables and reliance on subjective measurements for outcomes. Menzel-Severing et al. [21] reported severe risk in D4, with protocol inconsistencies that may jeopardize the overall reliability of their work. Williamson et al. [25] also had serious risk in D2, implying high methodological concerns over the control of confounders. Yip et al. [26] and Rozenberg et al. [23] reported moderate risks in D1, with bias due to confounding, and D3, respectively, which may have an impact on their conclusions.

### 3.8. Stratified and Subgroup Analyses

- Initiation Time (<12 h vs. >12 h)

When studies were reorganized according to initiation of HBOT before or after 12 h, a clear pattern emerged. The <12 h stratum generally favored HBOT, with repeated reports of meaningful visual improvement or better recovery trajectories, whereas the >12 h or variably delayed stratum showed attenuated, inconsistent, or statistically non-significant effects. This pattern aligned with the biologic rationale of retinal ischemic tolerance and explained an important portion of the between-study heterogeneity observed in the primary pooled analysis.

- Outcome Standardization (logMAR-Based Synthesis)

Standardization of visual outcomes around logMAR improved cross-study interpretability. Studies that already reported logMAR values, or those that provided data amenable to logMAR interpretation, facilitated clearer assessment of effect magnitude than studies reporting only decimal acuity, Snellen-line gains, or qualitative improvement. After harmonization, the overall direction of evidence remained favorable to early HBOT rather than being driven by any single reporting format. Nevertheless, incomplete reporting in several studies limited formal pooled analysis of continuous visual outcomes and necessitated narrative synthesis for part of the evidence base.

- Subgroup Analysis by Study Type

Subgrouping by study type showed that prospective and more structured comparative studies tended to provide more internally consistent evidence than retrospective case series. Prospective cohorts such as Chiabo et al. [17] and Shahzaib et al. [24] demonstrated coherent improvements in perfusion and visual acuity, while retrospective comparative studies such as Rozenberg et al. [23] and Lee et al. [15] suggested benefits but remained more vulnerable to confounding, selection effects, and variable treatment pathways. Smaller case series, including Lopes et al. [19] and Yip et al. [26], supported early HBOT but contributed greater imprecision. Thus, study design appeared to influence certainty of effect more than direction of effect.

- Subgroup Analysis by Baseline Severity

Baseline visual severity appeared to modify apparent treatment response, although reporting was incomplete and frequently non-standardized. Studies enrolling patients with very poor baseline visual acuity or profound ischemic presentations, such as those reporting baseline logMAR values above 2 or equivalent severe impairment, still documented measurable improvement after HBOT in some cohorts, indicating that severe presentation did not preclude benefit. However, the magnitude of recovery was heterogeneous, and comparisons across severity strata remained exploratory because baseline acuity was not uniformly reported for all studies. This incomplete severity reporting limits firm inference regarding whether HBOT performs better in moderate ischemia, profound vision loss, or only in selected subgroups.

- Residual Heterogeneity After Stratification

Even after stratification by timing, outcome metric, study type, and baseline severity, residual heterogeneity remained. Mixed CRAO/BRAO cohorts, combined therapies, variable comparator standards, and inconsistent follow-up durations continued to influence the pooled signal. Nonetheless, studies using standardized visual endpoints and clearly defined early treatment windows were more directionally concordant and generally produced lower unexplained heterogeneity than studies with broader eligibility and less structured outcome reporting.

- Early (<12 h) Treatment

The early-treatment subgroup included Beiran et al. [16], Lopes et al. [19], Menzel-Severing et al. [21], Shahzaib et al. [24], and comparative cohorts with early-weighted mean treatment initiation such as Rozenberg et al. [23]. Across this stratum, visual recovery was more consistently favorable, whether expressed as logMAR reduction, Snellen-line gain, or perfusion-associated acuity improvement. Shahzaib et al. [24] reported improvement from 20/200 to 20/80, Lopes et al. [19] reported improvement to a median BCVA of 0.7 logMAR, and Menzel-Severing et al. [21] documented a three-line Snellen gain. Collectively, these findings supported the clinical relevance of initiating HBOT within the early ischemic window.

- Delayed (>12 h) Intervention

The delayed-treatment subgroup comprised studies with median or mean initiation beyond 12 h or with treatment windows extending well beyond the early period, including Rosignoli et al. [22], Yip et al. [26], Lee et al. [15], Chiabo et al. [17], Maldonado et al. [20], and Kalaw et al. [18]. This stratum showed mixed effects: some studies still reported improvement, particularly when HBOT was delivered within 24 h, but the magnitude and consistency of the benefit were reduced relative to the early subgroup. Rosignoli et al. [22] and Kalaw et al. [18] showed little or no significant benefit, whereas Chiabo et al. [17] and Maldonado et al. [20] suggested that delayed but not excessively late treatment may retain partial efficacy. The >12 h stratum supported a time-response gradient rather than a uniform absence of effect.

## 4. Discussion

### 4.1. Thematic Findings from the Review

Across the included evidence, the most consistency was observed not simply in whether HBOT was used but when it was used and how outcome was measured. Reorganizing the evidence by initiation time and standardizing visual acuity interpretation around logMAR revealed that the benefit signal was concentrated in studies with earlier treatment and clearer outcome reporting. Studies such as Beiran et al. [16], Lopes et al. [19], Rozenberg et al. [23], Shahzaib et al. [24], and Yip et al. [26] supported a clinically relevant visual benefit, although the certainty of that benefit varied according to study design and baseline severity reporting.

By contrast, Kalaw et al. [18] and Rosignoli et al. [22] remained important counterweights because they reported limited or absent improvement despite HBOT exposure. These outlying results appeared to be linked more closely to delayed treatment windows, broader inclusion patterns, and methodological variability than to contradiction of the biological premise of HBOT itself. Accordingly, the present synthesis suggests that timing, study architecture, and baseline disease severity should be treated as core analytical strata in future CRAO evidence synthesis rather than secondary considerations.

### 4.2. Outcome Comparison with Established Literature

A number of other reviews [1,2,3,4,5,11,27,28,29,30] align with the present findings. Our revised synthesis places greater emphasis on the interaction between timing and standardized visual outcome assessment, showing that the apparent benefit of HBOT becomes more coherent when studies are interpreted through <12 h versus >12 h initiation strata and, where possible, a common logMAR framework. This is congruent with Celebi et al. [1] and Murphy-Lavoie et al. [5], both of whom emphasized the time-dependent nature of retinal salvage and the biological plausibility of early oxygen-based intervention.

At the same time, the present review extends the existing literature by showing that heterogeneity is not explained by timing alone. Variability in study design, mixed CRAO/BRAO case composition, and incomplete baseline severity reporting continued to affect interpretability even after outcome harmonization. This partly explains why more critical reviews, such as Sharma et al. [11] and Roskal-Wałek et al. [28], judged the evidence base to be insufficiently robust despite biologic plausibility and supportive single-center studies. Thus, the current evidence may be better interpreted as conditionally favorable for early HBOT rather than uniformly definitive across all CRAO presentations.

### 4.3. Timing and Visual Prognosis in CRAO

Time from symptom onset to therapy initiation emerged as the principal clinical modifier of visual prognosis after central retinal artery occlusion [28]. In the present stratified synthesis, the benefit signal was strongest in studies initiating HBOT within 12 h and became progressively less consistent once treatment crossed that threshold or was reported across broader time windows. Hyperbaric oxygen therapy likely acts by temporarily sustaining ischemic retina through choroidal oxygen diffusion; therefore, its effect is intrinsically linked to the duration of untreated ischemia.

### 4.4. Time to Therapeutic Intervention

There is substantial evidence that retinal ganglion cells are highly susceptible to hypoxia and that the window for salvage is narrow. Some reports, including Hertzog et al. [31] and Beiran et al. [16], emphasized treatment within 8 h, whereas Butler et al. [32] and the related ophthalmic literature suggested that meaningful benefit may still be achievable within 12 h. The present review supports a pragmatic interpretation: <12 h is the most clinically informative stratification threshold, while treatment beyond 12 h may still confer partial benefit in selected cases but with markedly greater uncertainty.

### 4.5. Evidence from Clinical and Meta-Analytic Studies

Many authors, such as Wu et al. [33], concluded that timing could not be statistically examined adequately because of heterogeneity in trial structure and reporting. The present review partially addressed that limitation by separating studies according to initiation time and by harmonizing visual endpoints toward logMAR whenever feasible. Even with these refinements, continuous pooled analysis remained restricted by incomplete reporting, which reinforces the need for future studies to publish convertible baseline and follow-up acuity data.

### 4.6. Variations in Timing Recommendations

Although most research concurs that early intervention is critical, recommendations still vary regarding the optimal window [34,35,36,37]. The current synthesis suggests that this variability partly reflects inconsistent reporting rather than purely biological disagreement. Once studies were interpreted through common timing strata and outcome criteria, the overall direction favored the earliest feasible HBOT initiation, while delays beyond 12–24 h were associated with a weaker and less predictable visual response.

### 4.7. Interdisciplinary Barriers to Timely Implementation of HBOT in CRAO

In spite of the encouraging evidence for early HBOT in CRAO, i.e., within the first 6 to 12 h of symptom onset [16,21,24,25], the practicability of the application of HBOT therapy in emergency clinical practice is severely limited by inter-specialty procedural demands. Anesthesiologists, the usual operators of HBOT chambers, usually require a chest radiograph before therapy initiation to rule out pneumothorax, in light of the risk of pulmonary barotrauma in a hyperbaric setting [5,30]. Moreover, otolaryngologic evaluation is routinely required to evaluate the risk for middle ear barotrauma, a recognized complication of pressurized oxygen environments [5,30]. These precautions, reasonable as they are, necessarily lead to delays in the commencement of therapy and may drive patients beyond the narrow therapeutic window demonstrated to be critical to retinal tissue salvage [16,21,24]. Moreover, neurologists usually diagnose CRAO as a stroke-equivalent event and require urgent neurovascular evaluation and transfer to stroke units prior to the potential consideration of ancillary therapy [5,11,28]. Such practice, while based upon the systemic nature of CRAO, imposes further delays that usually render HBOT impractical within the optimal treatment window. Thus, in spite of firm clinical precedent and supportive evidence for early HBOT in CRAO, these inter-specialty procedural barriers pose a strong deterrent to urgency in therapy, ultimately limiting the practical application of this treatment.

### 4.8. Limitations

A major limitation of this study was the considerable heterogeneity in study design, sample size, treatment timing, comparator strategy, and HBOT protocol. Although visual outcomes were standardized to logMAR whenever possible, several studies did not report sufficiently detailed convertible data, which limited quantitative synthesis of continuous outcomes. Baseline visual severity was also incompletely described across studies, restricting the robustness of severity-based subgroup analyses. The predominance of retrospective non-randomized designs further increased susceptibility to confounding and selection bias. Non-standardized adverse-event reporting may additionally have led to underestimation of safety signals.

### 4.9. Implications for Future Research

Future research should prioritize prospective, adequately powered studies with protocolized early referral pathways. Reporting standards should require exact time-to-treatment values, baseline and follow-up visual acuity in logMAR or convertible form and explicit stratification by baseline severity. Harmonization of chamber pressure, session number, and comparator treatment would facilitate more valid pooled analyses. Such design improvements would allow future meta-analyses to determine more definitively whether the benefit of HBOT is concentrated in specific timing windows or severity strata. Additionally, there is a need for randomized controlled trials (RCTs) to investigate the efficacy of HBOT so as to ascertain the efficacy of this modality.

## 5. Conclusions

HBOT showed the greatest promise for CRAO when initiated early, particularly within 12 h of symptom onset, while benefit after delayed treatment remained variable. Standardization of visual acuity outcomes around logMAR improved interpretability across studies, yet substantial heterogeneity persisted because of differences in study type, baseline severity reporting, and treatment protocols. HBOT therefore remains a potentially valuable adjunct in selected patients, but its role should be defined by better-timed, prospectively designed studies with uniform outcome criteria.

## Figures and Tables

**Table 1:** Studies included in the review and their observed demographic inferences.

**Study ID**	**Year**	**Location**	**Study Design**	**Sample Size**	**Mean Age (in Years)**	**Male: Female Ratio**	**Follow-Up Period**	**Groups Assessed**
**Lee et al.** [15]	2024	Korea	Comparative retrospective study	50	65.69	31:19	6 months	HBOT group (n = 29), Control group (n = 21)
**Beiran et al.** [16]	2001	Israel	Comparative retrospective study	72	69.5 ± 11.5	21:14	NA	HBO-treated vs. Non-HBO
**Chiabo et al.** [17]	2024	France	Prospective monocentric study	31	68.3 (15–93)	12:19	1 month	CRAO and BRAO
**Kalaw et al.** [18]	2024	United States	Retrospective cohort study	75	66.7	42:33	119.7 days	CRAO and BRAO
**Lopes et al.** [19]	2019	Portugal	Retrospective case series	13	70 (range 41–83)	08:05	NA	CRAO and BRAO
**Maldonado et al.** [20]	2024	Portugal	Retrospective study	114	69	77:37	Median 7 sessions	CRAO
**Menzel-Severing et al.** [21]	2012	Germany	Retrospective non-randomized case series	51	69 (range 35–82)	27:24	3 months	CRAO treated vs. control
**Rosignoli et al.** [22]	2022	USA	Retrospective chart review	48	69.3 ± 10.4	02:03	Variable	HBOT vs. No HBOT
**Rozenberg et al.** [23]	2021	Israel	Retrospective comparative analysis	144	69 ± 12 (HBOT), 60 ± 3 (SOC)	81:40 (HBOT), 17:6 (SOC)	Median 12.9 ± 34 months	HBOT vs. SOC
**Shahzaib et al.** [24]	2024	Pakistan	Prospective observational	85	55.67	52:33:	1 month	CRAO and BRAO patients
**Williamson et al.** [25]	2023	Australia	Retrospective analysis	22	64	15:07	NA	CRAO and BRAO
**Yip et al.** [26]	2020	Hong Kong	Retrospective case series	31	55.67 ± 4.81	17:08	Daily during treatment	CRAO patients

**Table 2:** Studies included in the review and their observed methodological and outcome inferences.

**Study ID**	**Primary Outcome Measure**	**Intervention Details**	**Timing of Intervention**	**Biochemical Markers Monitored**	**Visual Acuity Metrics**	**Adverse Events Recorded**	**Statistical Methods Used**	**Conclusion Assessed**
**Lee et al.** [15]	Change in visual acuity (BCVA) and OCT parameters	HBOT at 2.8 ATA, 100% oxygen, 90 min twice daily for 3 days, then 120 min once daily for 14 days	Within 7 days of symptom onset	None reported	logMAR at baseline and follow-up	None	Repeated-measures ANOVA, Dunnett’s post hoc test, Mann–Whitney U test, linear regression, Shapiro–Wilk test	HBOT significantly improved BCVA and preserved retinal and choroidal thickness compared to controls
**Beiran et al.** [16]	Change in Visual Acuity (VA)	HBOT: 2.8 ATA for 90 min	<8 h	None	0.2981 vs. 0.1308 mean VA (*p* < 0.03)	None significant	Chi-square, Wilcoxon test	Early HBO effective for hypertensive RAO patients
**Chiabo et al.** [17]	BCVA improvement ≥0.3 logMAR	HBOT at 2.5 ATA, 90 min, 2 sessions/day	Within 7 days of symptoms onset	Fluorescein angiography	1.51 to 1.1 logMAR (*p* < 0.05)	Minor barotrauma	I2 test, Student’s *t*-test	HBOT is effective and safe
**Kalaw et al.** [18]	BCVA change in logMAR	Emergency HBOT cycles	During emergency presentation	None	No significant change in BCVA	None reported	Linear mixed-effects models	HBOT did not improve BCVA outcomes significantly
**Lopes et al.** [19]	BCVA improvement	HBOT at 2.5 ATA for 90 min	Median 9 h (range 2–20)	None	Median BCVA improved to 0.7 logMAR	None reported	Wilcoxon signed-rank test (SPSS v22)	HBOT safe and effective if early treatment
**Maldonado et al.** [20]	Best-corrected visual acuity (BCVA)	HBOT at 2.4 ATA, 90 min sessions	Within 24 h of symptoms onset	None	Pre-HBOT BCVA: 2.12 ± 0.74; Post-HBOT: 1.67 ± 0.74	6% reported adverse events	Multiple linear regression, MANOVA	HBOT is safe and beneficial for early CRAO cases
**Menzel-Severing et al.** [21]	VA improvement (Snellen)	HBOT + hemodilution	Within 12 h of symptoms onset	None	3 lines improvement (*p* < 0.0001)	None	Chi-square, linear regression	Combined treatment shows VA improvement
**Rosignoli et al.** [22]	VA improvement (logMAR)	HBOT using US Navy diving protocol	Variable, median 18.27 h	None	No statistical significance (*p* = 0.83)	Barotrauma, seizures	Chi-square, Student’s *t*-test	HBOT not effective for CRAO
**Rozenberg et al.** [23]	Best Corrected Visual Acuity (LogMAR)	HBOT: 2.4 ATA for 90 min (initial), 2 ATA (subsequent); SOC: ocular massage, meds	Mean 9.1 ± 5 h post-onset (HBOT)	None	Improved from 2.89 ± 0.98 to 2.15 ± 1.07 LogMAR	None reported	*t*-test, Mann–Whitney U, logistic regression (SPSS v24)	HBOT improves VA vs. SOC significantly
**Shahzaib et al.** [24]	Best Corrected Visual Acuity (BCVA)	HBOT at 2–2.5 ATA for 60–90 min per session	10 h (range 4–22)	Arterial filling time via fluorescein angiography	20/200 to 20/80 in 1 week	None reported	Comparison via SPSS v29	HBOT improves perfusion and VA when early
**Williamson et al.** [25]	Change in LogMAR BCVA	RBWH CRAO protocol: HBOT cycles	Median 12 h for CRAO	None	LogMAR improvement by −0.2 for CRAO	Hemotympanum, anxiety	Multiple linear regression	HBOT beneficial for CRAO, not for BRAO
**Yip et al.** [26]	Change in visual acuity (LogMAR)	HBOT at 2.8 ATA (initial), 2.4 ATA (subsequent)	Mean 13.3 ± 7.4 h post-onset	None	−0.43 LogMAR (*p* = 0.003)	None significant	Fisher’s exact test, *t*-test (SPSS v25)	HBOT shows promising outcomes

## Data Availability

All data underlying this study are derived from publicly available journal articles.

## Supplementary Materials

The following supporting information can be downloaded at: https://www.mdpi.com/article/10.3390/jcm15093530/s1, File S1: Prisma 2020 Checklist.

## Abbreviations

The following abbreviations are used in this manuscript:

HBOT	Hyperbaric Oxygen Therapy
CRAO	Central Retinal Artery Occlusion
